# Pilot Study of Genetic Diversity and Structure in Elite Germplasm of *Hibiscus syriacus*

**DOI:** 10.3390/plants14182909

**Published:** 2025-09-19

**Authors:** Yan Gao, Wei Yan, Chunying Zhang

**Affiliations:** Shanghai Botanical Garden, Shanghai Engineering Research Center of Sustainable Plant Innovation, Shanghai 200231, China

**Keywords:** *Hibiscus syriacus*, genetic diversity and structure, SSR, ISSR

## Abstract

Rose of Sharon (*Hibiscus syriacus* L.) is an important perennial deciduous ornamental plant, featured by the daily flowering habit and a prolonged flowering period. However, the genetic relationships of the elite germplasmare largely unclear, which hampers the breeding programs of *H. syriacus*. Here, we analyzed the genetic diversity andstructure of 46 cultivars by employing a combination of 10 simple sequence repeat (SSR) and 5 inter-simple sequence repeat (ISSR) polymorphicmarkers. On average, 1.251 effective alleles per locus were detected for the SSR markers, in contrast to 1.321 for ISSR. Consistently, these elite accessions were grouped into five clades when using either marker or a combination of both, albeit with some differences. In the combined topology, clade II contains three relatively less multiple-petaled accessions, “Notwoodone” and its branch mutant “Bricutts”, as well as *H. syriacus* var. *Shigyoku*. By contrast, “Duc de Brabant” and “Mindour1” are both pink multiple-petaled accessions in clade III, in addition to a solo single-petaled “Oiseau Bleu” in clade I. Clade V was the largest group of 34 accessions, which account for 73.9% of the evaluated *Hibiscus* varieties and cluster into six subclasses. Overall, these varieties have some morphological variances in both patterns and colors of flowers. They show similarities in subclass scale, as exemplified by “Lady Stanley” and its branch mutant, “America Irene Scott”. The distantly related varieties, like in clade I and clade V, would benefit for breeding new varieties of high-hybrid vigor. Together, we updated a pilot study of the genetic diversity andstructure in elite varieties of *H. syriacus*, which could provide new insights into marker-assisted selection and genetic breeding of new varieties.

## 1. Introduction

*Hibiscus syriacus* L., a perennial deciduous shrub in the Malvaceae family, is commonly known as Rose of Sharon [1]. It is historically and literarily referred to as the “Dawn Blossoms Plucked at Dusk” flower in Chinese due to its unique daily flowering habit, where individuals bloom in the early morning and fall by night [2]. However, it has a prolonged flowering period of about 6 months, typically from May to October in eastern Asia [3]. *H. syriacus* varieties are widely cultivated ornamental plants in North America and Eastern Asia, with an origin in China [4,5,6,7]. It also has highly edible value of rich nutritional components and inorganic elements in its leaves and petals [8], thus leading to a long-standing tradition of consumption worldwide. Moreover, *H. syriacus* is a medicinal plant, having the functions of detoxification and reducing swelling. Specifically, the water-soluble mucilage extracted from its leaves possesses both hypoglycemic and anti-mycobacterial activities [9,10]. Although it is native to China, limited progress has been achieved on genetic improvement and breeding in *H. syriacus*. Recently, numerous varieties have been introduced from abroad, yet the cultivation of *H. syriacus* faces significant challenges due to the confusion over its cultivar names that are not related to the genetic relationships and/or unclear genetic backgrounds [11]. Therefore, it is urgent to discriminate the genetic diversity of the elite *H. syriacus* variates and standardize the germplasm collections for application.

Thus far, genetic markers have endured four distinct stages, including morphological, cytological, biochemical, and molecular markers. Accordingly, studies on the genetic diversity analysis of *H. syriacus* have progressively transitioned from morphological to molecular markers. Based on the key morphological traits, including the red center of the petal, petal index, and the relationship between the red center line and the red center, the 27 *Hibiscus* cultivars were largely divided into three groups, in which “Woodbridge”, “Rubis”, and “Red Heart” gather together with similar morphological features [12]. Moreover, evidence indicated that *Paeoniflorus*, “Elegantissimus”, and “Arang” are probably the same, but with confused commercial names. Amplified fragment length polymorphism (AFLP) marker analysis of three winter-hardy *Hibiscus* species native to China revealed that *H. sinosyriacus* appears to be an intermediate form between *H. paramutabilis* and *H. syriacus* [13]. A total of 10pairs of sequence-related amplified polymorphism (SRAP) markers were applied to analyze the relationships of 39 *Hibiscus* cultivars and found that they were classified into five groups, with a reasonable observation that the genetic distances between different varieties of *Hibiscus* are much larger than those between different cultivars [14]. A large scale of expressed sequence tag-simple sequence repeat(EST-SSR) markers were developed from diverse transcriptomes of *H. syriacus* flower, stem, and leaf, which correspondingly generate 14.39%, 14.24%, and 13.99% SSR sequences when quantified by the total number of Unigenes, respectively [15]. Furthermore, 10pairs of high polymorphic SSR markers were used to construct DNA fingerprints of 15 *Hibiscus* cultivars. The genetic diversity of 41 *H. syriacus* varieties was characterized by using nine pairs of polymorphic AFLP markers and was similarly divided into five groups with an Nei index ranging from 1.18 to 1.25; however, some varieties of the same origin did not strictly cluster together [16]. These results suggested that the *H. syriacus* germplasm exhibits significant genetic variation and rich genetic diversity. In addition, five pairs of inter-simple sequence repeat (ISSR) markers were used to analyze the genetic relationships between *H. schizopetalus* and four cultivated varieties of *H. rosa-sinensis* and found that they share a high genetic similarity, supporting the notion that *H. schizopetalus* is a variety of *H. rosa-sinensis* [17]. Notably, the cultivar “Scarlet” has both amplified fragments that are unique to *H. rosa-sinensis* and *H. schizopetalus*, respectively, suggesting it is a hybrid origin.

Nonetheless, the genetic diversity and structure of the elite germplasm in *H. syriacus* remain to be further elucidated. In the present study, we combined SSR and ISSR markers to analyze the genetic diversity and phylogenetic relationships of 46 elite varieties in *H. syriacus*. Our findings could enrich and enhance the current understanding of the phylogenetic topology of *H. syriacus* cultivars, thus providing a genetic basis for the systematic classification of *H. syriacus* germplasm and the potential utilization in marker-assisted selection and breeding programs, like maximizing hybrid vigor and pyramiding multiple genes controlling the important traits.

## 2. Results

### 2.1. Polymorphism Detection of SSR and ISSR Markers

**Figure 1:**
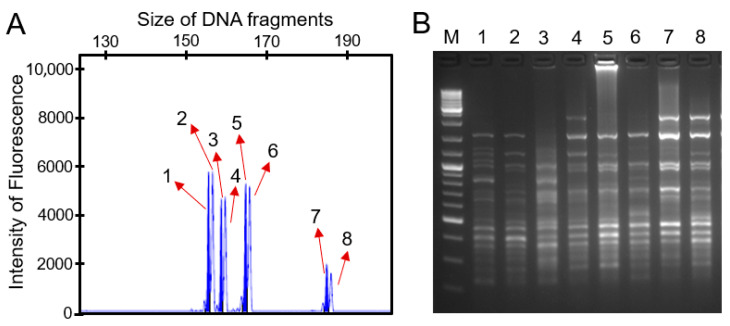
Polymorphism detection of SSR and ISSR markers used in *H. syriacus* varieties. (**A**) Peak graphs generated from fluorescence capillary electrophoresis of *H. syriacus* sample amplified using the marker HP0003. This visual representation highlights the diversity of fragment sizes detected, indicative of high polymorphism among the samples. Each peak corresponds to a specific amplified DNA fragment, with the height of the peak reflecting the abundance of each fragment within the sample (red arrow with number). (**B**) PCR products of *H. syriacus* samples amplified using the ISSR marker UBC840. Lane: 1, ROUFENHONGXIN; 2, *H. syriacus* f. totus-albus; 3, “Notwoodone”; 4, *H. syriacus* f. albus-plenus; 5, “Ds01bs”; 6, QIANSIBAN; 7, “Ardens”; and 8, “Admiral Dewey”.

To assess the genetic diversity within *H. syriacus* varieties, 10 pairs of SSR primers selected from a panel of 59 pairs were utilized to detect the DNA polymorphisms by separating the amplified products using fluorescence capillary electrophoresis. The results indicated that 7out of the 10SSR markers were polymorphic. A total of 33 amplified DNA fragments were obtained, of which 28 were polymorphic fragments that accounts for 84.85% of the whole (Table 1). On average, 2.8 polymorphic fragments were obtained for each maker. Of note, marker HP0003 demonstrated the highest variability, amplifying up to eight polymorphic fragments (Figure 1A).

In parallel, five selected pairs of ISSR markers were used, thus resulting in the amplification of 95 fragments, in which 69 were polymorphic, with an average polymorphism rate of 72.6% (Table 2). In contrast to the SSR markers, the average polymorphic fragments of each locus increased to 13.8. Likewise, the marker UBC840 generated 17 polymorphic fragments (Figure 1B). Therefore, the above 10SSR and 5ISSR markers were used to analyze the genetic diversity of the elite varieties in *H. syriacus*.

### 2.2. Evaluation of Genetic Parameters of 46 Elite H. syriacus Varieties Using the Polymorphic SSR and ISSR Markers

Genetic diversity analysis was in turn carried out on the 46 elite *H. syriacus* cultivars globally collected, using the polymorphic SSR and ISSR markers, respectively (Table 3). For SSR markers, the results demonstrated that the average observed number of alleles (Na) was 1.818, and the number of effective alleles (Ne) was 1.251, across the 10 detected loci. Shannon’s information index (I) was 0.163, while Nei’s gene diversity index (H) was 0.267. Consistently, the largest value of H was observed at HP0003 locus. By contrast, the results of the ISSR markers showed an average value of 1.674 for Na and1.321 for Ne. The corresponding values of Shannon’s information index (I) and Nei’s gene diversity index (H) were 0.305 and 0.197, respectively.

Combined together, these data indicate that the analyzed 46 elite *H. syriacus* varieties exhibit significant genetic variation and rich gene diversity. Intriguingly, the mean Na of SSR loci was higher than that of ISSR, whereas the mean Ne value was inversely higher at the ISSR relative to the SSR loci (Table 3). Consequently, genetic diversity was relatively greater at the ISSR loci than at the SSR loci, as evidenced by higher values of Nei’s gene diversity index and Shannon’s information index. Therefore, the genetic parameter, mean value of Ne, rather than Na, is the determining factor of genetic diversity analysis.

### 2.3. Phylogenetic Analysis of 46 Elite H. syriacus Varieties Based on the SSR Markers

The genetic similarity coefficients were calculated for the 46 *H. syriacus* cultivars by using the statistical method proposed by Neiet al. (1973) [18], resulting in a total of 1035genetic similarity coefficients that ranged from 0.485 to 1.0, with an average value of 0.836. The lowest coefficient, 0.485, was observed between *H. syriacus* f. *elegantissimus* and “Mindour1”, indicating their most distant genetic relationship within the population. Conversely, some of the varieties, such as“Rwoods6” and *H. syriacus* f. *albus-plenus*, exhibited a genetic similarity coefficient of 1.0, suggesting that they have a high genetic similarity. It should be pointed out that the resolution of the SSR markers in this study is not enough to discriminate between these similar varieties.

**Figure 2:**
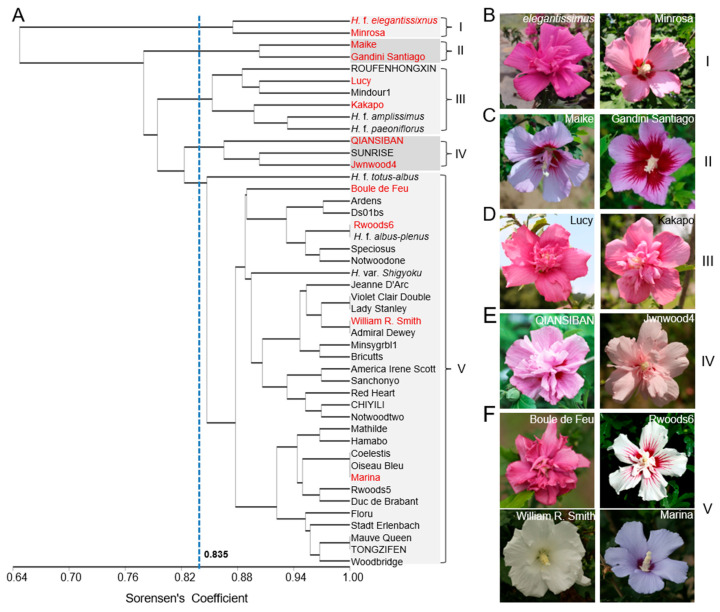
The UPGMA phylogenetic tree of 46 *H. syriacus* varieties based on SSR markers. (**A**) The topology resulted in five clades under a genetic similarity coefficient threshold of 0.835. (**B**–**F**) Representative *H. syriacus* varieties in clade I **(B**), II (**C**), III (**D**), IV (**E**), and V (**F**). The varieties in red are the representative ones.

Following UPGMA cluster analysis, a phylogenetic tree was constructed, as depicted in Figure 2. Using a genetic similarity coefficient threshold of 0.835, the 46 elite varieties were divided into five clades (Figure 2A). Of note, clade I and II were the smallest groups, both of which contained two members. *H. syriacus* f. *elegantissimus* and “Minrosa” in clade Iare characterized by dark-pink flower pigmentation, but with an unexpectedly different petal pattern (Figure 2B). By contrast, clade II comprised “Maike” and “Gandini Santiago”, which have single-petaled, light-pink flowers with purple radial lines in the center (Figure 2C). Clade III contained six members, such as ROUFENHONGXIN, “Lucy”, “Mindour1”, “Kakapo”, *H. syriacus* f. *amplissimus*, and *H. syriacus* f. *paeoniflorus*, which are all multiple-petaled varieties with colors varying from light pink to red (Figure 2D). Clade IV included QIANSIBAN, RICHUHONG, and “Jwnwood4”, all exhibiting light pink to pink colors (Figure 2E). Clade V was the largest group comprising 33 varieties, such as “Boule de Feu”, *H. syriacus* f. *totus-albus*, “William R. Smith”, and “Marina” (Figure 2F). This clustering effectively grouped the elite germplasm with identical genetic similarity coefficients and delineated five distinct groups. Notably, some certain variety pairs with a similarity coefficient of 1.0include“Rwoods6” and *H. syriacus* f. *albus-plenus, “Violet* Clair Double” and “Lady Stanley”, “William R. Smith” and “Admiral Dewey”, “Coelestis” and “Oiseau Bleu” and “Marina”, “Mauve Queen” and TONGZIFEN, which indicates indistinguishable differences between them or they could not be differentiated under the current resolution.

### 2.4. Phylogenetic Analysis of 46 Elite H. syriacus Varieties Based on the ISSR Markers

**Figure 3:**
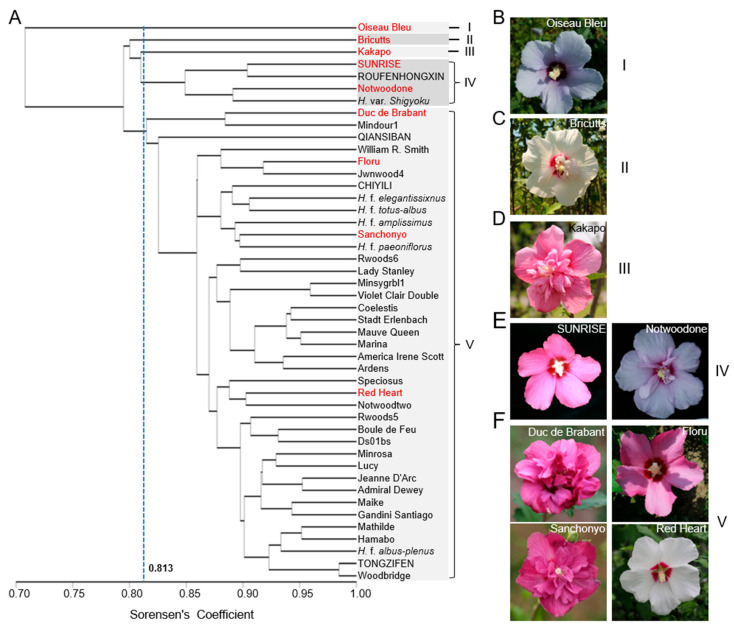
The UPGMA phylogenetic tree of 46 *H. syriacus* varieties based on ISSR markers. (**A**) The phylogenetic tree resulted in five clades under a genetic similarity coefficient threshold of 0.813. (**B**–**F**) Representative *H. syriacus* varieties in clade I (**B**), II (**C**), III (**D**), IV (**E**), and V (**F**). The varieties in red are the representative ones.

Similarly, a comprehensive analysis of the ISSR markers was performed for the above 46 *H. syriacus varieties*, overall yielding 1035 genetic similarity coefficients, which ranged from 0.635 to 0.985, with an average value of 0.842. Among them, “Oiseau Bleu” and “Admiral Dewey” demonstrated the most distant genetic relationship (a value of 0.635), whereas “Woodbridge” and “TONGZIFEN” exhibited the closest relationship (a value of 0.985). UPGMA clustering analysis led to a kinship dendrogram, as depicted in Figure 3A, which classified the 46 varieties into five groups at a similarity coefficient threshold of 0.813.Notably, “Oiseau Bleu”, “Bricutts”, and “Kakapo” were clustered individually, with each into a single clade, indicating that they are not only distant from each other, but also have significant genetic distances from the rest ones. Indeed, they display drastic morphological changes, in which “Oiseau Bleu” is a typical single-petaled light-purple flower variety (Figure 3B), and “Bricutts” flowers are white but slightly multiple-petaled with a red center (Figure 3C), in comparison to the pink multiple-petaled “Kakapo” (Figure 3D). Clade IV contained four members, as exemplified by RUNRISE and “Notwoodone”, which are slightly multiple-petaled varieties (Figure 3E).The remaining 39 elite lines formed the largest clade V, which were mingled by diverse flower colors and patterns (Figure 3F).

Moreover, comparison of the two phylogenetic trees revealed distinct clustering outcomes between SSR and ISSR markers, highlighting some differences in the genetic structures detected by these two DNA makers. In both cases, clade V is the largest group, comprising of 29 overlapping varieties, which accounted for 87.88% and 74.36% of the total in the SSR and ISSR marker-based topologies, respectively. Nonetheless, some divergences were indeed observed between them, which are speculated to arise from the different polymorphism ratios and numbers of the two kinds of markers.

### 2.5. Phylogenetic Analysis of 46 Elite H. syriacus Varieties Based on the Combined SSR and ISSR Markers

To rule out individual differences, a combined analysis of the SSR and ISSR markers was performed, and a total of 1035 genetic similarity coefficients were obtained between pairs of the 46 *H. syriacus varieties*. The coefficients ranged from 0.682 to 0.981, with an average value of 0.841, indicating rich genetic diversity within the collections. Among them, “Oiseau Bleu” exhibited the most distant genetic relationships to “Minrosa” and *H*. *syriacus* f. *elegantissimus*, while “Woodbridge” and TONGZIFEN were the closest pair.

**Figure 4:**
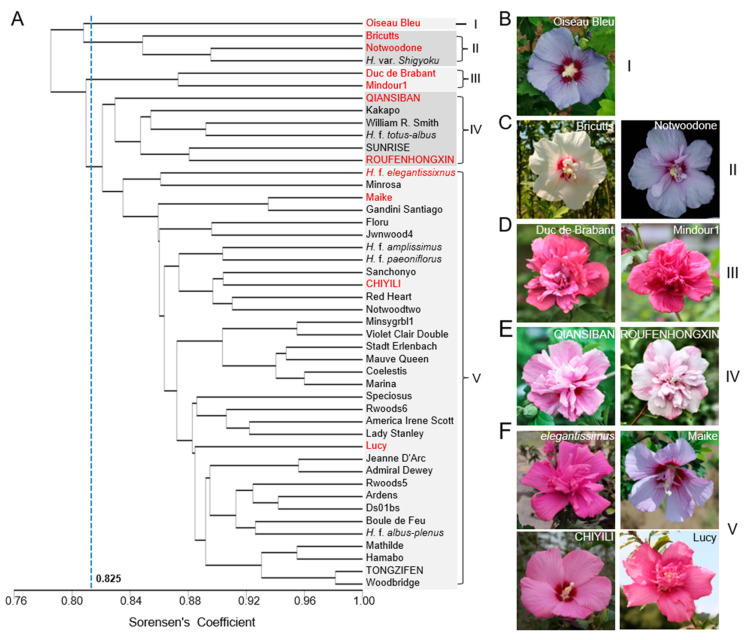
The UPGMA phylogenetic tree of 46 *H. syriacus* varieties based on the combined SSR and ISSR markers. (**A**) The topology resulted in five clades under a genetic similarity coefficient threshold of 0.825. (**B**–**F**) Representative *H. syriacus* varieties in clade I (**B**), II (**C**), III (**D**), IV (**E**), and V (**F**). The varieties in red are the representative ones.

A phylogenetic tree was further constructed by the integration of SSR and ISSR marker data (Figure 4A). The topology categorized the 46 varieties into five distinct clades based on a similarity coefficient threshold of 0.825. Similarly, “Oiseau Bleu” was notably isolated individually, forming a single clade (Figure 4B). Three relatively less multiple-petaled accessions, “Bricutts”, “Notwoodone”, and *H. syriacus* var. *Shigyoku*, comprised clade II (Figure 4C). Of note, “Bricutts” is a novel branch mutation of “Notwoodone”. Clade III contained “Duc de Brabant” and “Mindour1”, both of which are dark-pink multiple-petaled (Figure 4D). By contrast, clade IVis comprised of six members, some of which are light-pink multiple-petaled varieties, such as QIANSIBAN and ROUFENHONGXIN (Figure 4E). In agreement, clade V was the largest group containing 34 members that account for 73.9% of the elite varieties and could further divide into six subclasses. Unsurprisingly, morphological variances in both patterns and colors of flowers were observed in these varieties (Figure 4F). However, they are highly similar at the subclass level, such as “Lady Stanley” and its branch mutant, “America Irene Scott”.

## 3. Discussion

Genetic diversity reflects the range of genetic variation among individuals within a species or a group of collections. More attention has been paid toward germplasm resources and genetic variance of *Hibiscus* species, as analyzed by markers at morphological, cytological, biochemical, and DNA levels. In this study, 10pairs of SSR markers were selected based on apreviousreport [15], and 7exhibited ideal polymorphisms, making them particularly suitable for assessing the genetic diversity of *H. syriacus* varieties. However, these SSR markers were insufficient to fully differentiate the46 analyzed elite germplasm. For example, the genetic similarity coefficient among “Coelestis”, “Oiseau Bleu”, and “Marina” was 1.0, indicating that these three phenotypically light-blue varieties appear to be closely related, but indeed not identical to each other. We assumed that the limited resolution of the SSR markers likely stems from their inherent nature, which relies on simple sequence repeats and often results in relatively low polymorphism. This hypothesis is in line with the finding from similar studies on *Phaseolus vulgaris* [19].To overcome this limitation, it is recommended to add the number of SSR markers and/or to utilize a combination of multiple genetic markers. This approach would allow for more effective and accurate analysis of genetic diversity in *H. syriacus* varieties, helping standardize the germplasm collections.

ISSR markers were therefore used to detect genetic diversity of the varieties. Comparison of the clustering results of the two analyses revealed overall similarities and also some differences. For example, “Woodbridge” and TONGZIFEN were consistently identified as closely related members in both analyses. However, discrepancies were also observed between the SSR and ISSR results. For example, in the ISSR analysis, “Oiseau Bleu” and “Bricutts” were clustered individually, whereas in the SSR result, these two varieties were grouped together with the other 31 members in clade V. These differences are speculated to have arisen from the varying polymorphism ratios yielded bydifferent molecular markers and also the insufficient number of markers used. In essence, SSRs and ISSRs are locus-specific markers that are highly abundant in the genome, in which the former are inherited in a co-dominant pattern relative to a dominant mode of the latter, thus strongly affecting the informativeness and reproducibility [20]. This limitation may prevent the accuracy of genetic diversity analysis in *Hibiscus* resources. Moreover, it is crucial to recognize that phenotypic plasticity is a complex process that depends on interactions between individual organisms and the changeable environmental conditions [21]. This scenario highlights the inherent complexity of accurately interpreting phenotypic traits that are based solely on molecular data. An alternative explanation could be that some morphological traits, such as flower color, might be controlled by some other genes that are not linked to the markers used in our study.

The phylogenetic tree, constructed by the combined SSR and ISSR markers, successfully categorized the 46 varieties into five distinct clades. Prominently,“Oiseau Bleu”, the early light-blue single-petaled variety, is individually separated as a single clade. In clade II, all three varieties are slightly multiple-petaled, and “Bricutts” is originated from a branch mutation of “Notwoodone”. Likewise, “Duc de Brabant” and “Mindour1” in clade III are highly similar in flower color and pattern, albeit with slight difference in flower size. However, it is challenging to distinguish the members in clade IVand clade V when only based on flower colors and patterns. It is should be pointed out that they also have differences in detailed flower traits, such as eye spots and radial lines, and vegetative traits. Further, the geographic origins of the varieties span different ecological regions [1], which suggests that other uncharacterized features also contribute to their grouping. It is thus important to increase the available number of SSR and ISSR markers to enhance the resolution of the phylogenetic topology of the elite varieties, making a more accurate link between the phenotype and the genotype.

The application of SSR and ISSR molecular markers, either sole or combined, revealed that different molecular markers exhibit varying degrees of detected polymorphism. This finding is consistent with the result from the genetic diversity analysis of 12 *Elymus* species using SSR and ISSR markers [19,22]. In this study, 10pairs of SSR primers and 5 pairs of ISSR primers were utilized to analyze the genetic diversity of 46 *H. syriacus* varieties, revealing high levels of polymorphism for both sets of primers. The integration of SSR and ISSR markers provided more reliable clustering results, largely reflecting the genetic relationships of these resources. Nonetheless, the genetic diversity identified did not fully correspond to the morphological characteristics of the cultivars. The UPGMA cluster analysis illuminated the genetic relationships of these varieties, yet no consistent morphological features were observed across different clusters. This result is consistent with the finding from the study on the genetic relationships analysis in *Loropetalumchinense* var. *rubrum* cultivars [23]. In addition, discrepancies between clustering results obtained through single versus combined maker analyses were found, which is similar to that reported in genetic diversity of radish (*Raphanus sativus* L.) using ISSR and SSR markers [24]. Two major factors likely contribute to these discrepancies. Firstly, the resolution of SSR and ISSR markers may be insufficient to precisely distinguish all varieties. Secondly, molecular markers reveal genetic differences at the molecular level, while phenotypic traits arise from interactions between genetic and environmental factors. The findings of this study hold significant value for the conservation and utilization of *Hibiscus* resources and provide a pilot basis for future efforts in marker-assisted selection and genetic breeding of new varieties. For instance, novel varieties could be created by crossing distantly related ones at the clade and subclade levels by increasing the hybrid vigor and complementing beneficial traits.

## 4. Materials and Methods

### 4.1. Plant Material

The 46 *H. syriacus* varieties world-widely distributed and applicated (Table 4) were used for genetic diversity analysis. The materials include seven variants or cultivars from China, while the other 39 varieties are obtained from Japan, Europe, and the United States, as well as many unknown regions. For example, the Chiffon series of *Hibiscus*, here includes six cultivars, such as “Notwoodtwo”, “Notwoodone”, “Jwnwood4”, “Rwoods5”, “Rwoods6”, and “Bricutts”, which are a cultivar series hybridized with British horticulturist Roderick Woods from the United Kingdom.“Ds01bs” is an American cultivar with multiple petals. Japanese cultivars include *H. syriacus* v. *Shigyoku*, SUNRISE, and CHIYILI. The “Woodbridge” variety is a classic cultivar hybridized with the British Nottcutts Nursery. The Dutch “Pillar” series of *Hibiscus* are fast-growing and have a compact plant architecture and excellent erectness. Additionally, there is one variegated cultivar from Missouri, USA, named “America Irene Scott”, which is a branch mutation of “Lady Stanley”. Some cultivars have unknown genetic backgrounds due to the lack of detailed breeding records or unknown open-pollinated parents.

### 4.2. DNA Extraction

The samples were taken from *Hibiscus* resource garden of Shanghai Botanical Garden. Briefly, four young leaves were collected from each cultivar and were quickly frozen using liquid nitrogen. Leaf DNA was extracted using a plant tissue genomic DNA extraction kit from Sangon Biotech (Shanghai, China). DNA integrity was analyzed by using agarose gel electrophoresis. DNA concentration and purity was determined using a SMA4000 spectrophotometer (Merinton nstrument, Beijing, China). Gel imaging system recorded imaging information to detect the degree of DNA completion. Dilute qualified DNA with double-distilled water to a concentration of 50 ng·μL^−1^ and stored at −20 °C until use.

### 4.3. PCR Amplification

A total of 10 pairs of SSR primers were used as described before [15], as listed in Table 5. A total of 100 universal ISSR primer sequences developed by UBC (University of British Columbia) in 2006 were pre-experimented. Primers with clear bands, high repeatability, and formation of 5–11 polymorphic bands were selected for further analysis (Table 2). The PCR reaction includes 2.5 μL of 10 X PCR buffer, 2.5 μL of 2 mM dNTP, 0.5 μL of 10 µM primer (each), 0.2 μL of Taq Plus DNA Polymerase (5 U/μL), and 100 ng DNA template and add water into 25 μL. The PCR procedure for SSR marker and ISSR is as described before [15,19,22]. Briefly, the SSR procedure is as following: pre-denature at 95 °C for 5 min, a first 10 cycles (denature at 94 °C for 30 s, annealing at 60 °C for 30 s with a decrease of 0.5 °C per cycle, and extension at 72 °C for 30 s), a second 30 cycles (denature at 94 °C for 30 s, annealing at 55 °C for 30 s, and extension at 72 °C for 30 s), and a final extension at 72 °C for 10 min; the ISSR is as following parameters: pre-denature at 94 °C for 5 min, 35 cycles (denature at 94 °C for 30 s, annealing at 55–60 °C for 30 s for each primer, and extension at 72 °C for 3 min), and a final extension at 72 °C for 10 min.

### 4.4. Statistical Analysis of Data

PCR amplification products are separated by 1.2% agarose gel and imaged under the BIO-RAD DOC-2000 Automated Gel Imaging System. Briefly, the results of the electrophoresis spectrum were counted; the clear, stable, and reproducible bands were selected as “1”, and the weak or no bands that were not repeated at the same position were marked as “0” to form a “0.1” matrix.Gene mapper v4.0 (Applied Biosystems, Foster City, CA, USA) is used to read ISSR and SSR data and analyze genetic diversity indices such as genetic distance and genetic similarity coefficient. MVSP3.13 is used to analyze Hibiscus cultivars according to UPGMA (unweighted pair–group method with arithmetic means, [25]). Phylogenetic tree is constructed to realize the genetic relationship between *Hibiscus* varieties.

## Figures and Tables

**Table 1:** SSR markers and the amplification results in *H. syriacus* samples.

Primer Name	Num. of Amplified Bands	Num. of Nonpolymorphic Bands	Num. of Polymorphic Bands	Polymorphic Ratio (%)
HP0001	2	1	1	50.00
HP0002	1	1	0	0.00
HP0003	8	0	8	100.00
HP0010	2	2	0	0.00
HP0011	3	0	3	100.00
HP0012	4	0	4	100.00
HP0015	5	0	5	100.00
HP0018	4	0	4	100.00
HP0038	3	0	3	100.00
HP0055	1	1	0	0.00
Total	33	5	28	
Average	3.3	0.5	2.8	84.85

**Table 2:** ISSR markers and the amplification results in *H. syriacus* samples.

Primer Name	Sequence	Num. of Amplified Bands	Num. of Nonpolymorphic Bands	Num. of Polymorphic Bands	Polymorphic Ratio (%)
UBC809	AGA GAG AGA GAG AGA GG	20	8	12	60.00
UBC827	ACA CAC ACA CAC ACA CG	16	5	11	68.75
UBC836	AGA GAG AGA GAG AGA GYA	20	5	15	75.00
UBC840	GAG AGA GAG AGA GAG AYT	20	3	17	85.00
UBC842	GAG AGA GAG AGA GAG AYG	19	5	14	73.68
Total		95	26	69	
Average		19	5.2	13.8	72.63

**Table 3:** Genetic parameters of 46 elite *H. syriacus* varieties evaluated by using the polymorphic SSR and ISSR markers.

Marker	Item	Number of Primers	Na (Observed Number of Alleles)	Ne (Effective Number of Alleles)	H (Nei’s Gene Diversity Index)	I (Shannon Information Index)
SSR	Mean value	10	1.818	1.251	0.163	0.267
	Standard deviation		0.068	0.053	0.028	0.040
ISSR	Mean value	5	1.674	1.321	0.197	0.305
	Standard deviation		0.053	0.035	0.019	0.027

**Table 4:** Details of the 46 elite *H. syriacus* varieties.

No.	Variety Name	Flower Pattern	Color	Eye Spot (Y/N)	Country
1	*H*. *syriacus* “Woodbridge”	Single	Deep Pink	Y	UK
2	*H*. *syriacus* “Notwoodtwo”	Semi-double	White	N	UK
3	*H*. *syriacus* f. *paeoniflorus*	Double	Pink	Y	China
4	*H*. *syriacus* TONGZIFEN	Single	Orchid Pink	Y	China
5	*H*. *syriacus* “Gandini Santiago”	Semi-double/Single	Mauve Pink	Y	Netherlands
6	*H*. *syriacus* “MINDOUR 1”	Double	Purple-Red	Y	France
7	*H*. *syriacus* “Red Heart”	Single	White	Y	unknown
8	*H*. *syriacus* “Jwnwood4”	Semi-double	Pink	Y	UK
9	*H*. *syriacus* “Duc de Brabant”	Double	Rose-Purple	Y	unknown
10	*H*. *syriacus* var. *Shigyoku*	Double	Purple	Y	Japan
11	*H*. *syriacus* f. *amplissimus*	Double	Pink-Purple	Y	China
12	*H*. *syriacus* “Bricutts”	Semi-double	White	Y	UK
13	*H*. *syriacus* ROUFENHONGXIN	Double	Pink	Y	China
14	*H*. *syriacus* f. *totus-albus*	Single	White	N	China
15	*H*. *syriacus* “Notwoodone”	Semi-double	Lavender	Y	UK
16	*H*. *syriacus* f. *albus-plenus*	Double	White	Y	China
17	*H*. *syriacus* “Ds01bs”	Double	Blue-Purple	Y	US
18	*H*. *syriacus* QIANSIBAN	Double	Pink	Y	China
19	*H*. *syriacus* “Ardens”	Double	LilacPurple	Y	unknown
20	*H*. *syriacus* “Admiral Dewey”	Double	White	N	unknown
21	*H*. *syriacus* “Boule de Feu”	Double	RosyRed	Y	unknown
22	*H*. *syriacus* “Hamabo”	Single	BlushPink	Y	unknown
23	*H*. *syriacus* “Lady Stanley”	Double	Pink	Y	unknown
24	*H*. *syriacus* “Rwoods5”	Double	Red-Purple	Y	UK
25	*H*. *syriacus* ‘Maike’	Semi-double	Pink-Purple	Y	unknown
26	*H*. *syriacus* “Marina”	Single	Blue	Y	Netherlands
27	*H*. *syriacus* “Mathilde”	Single	Pink	Y	Netherlands
28	*H*. *syriacus* “Mauve Queen”	Single	Red-Purple	Y	US
29	*H*. *syriacus* “Oiseau Bleu”	Single	Sky Blue	Y	France
30	*H*. *syriacus* CHIYILI	Single	Red-Purple	Y	Japan
31	*H*. *syriacus* “Sanchonyo”	Double	Red-Purple	Y	unknown
32	*H*. *syriacus* “Jeanne D’Arc”	Double	White	N	unknown
33	*H*. *syriacus* RUNRISE	Single	Red	Y	Japan
34	*H*. *syriacus* “William R. Smith”	Single/Semi-double	White	N	US
35	*H*. *syriacus* “Rwoods6”	Semi-double	White	Y	UK
36	*H*. *syriacus* “America Irene Scott”	Double	Pink	Y	US
37	*H*. *syriacus* “Stadt Erlenbach”	Semi-double/Single	Lilac	Y	unknown
38	*H*. *syriacus* “Floru”	Single	Red-Purple	Y	France
39	*H*. *syriacus* “Violet Clair Double”	Double	Purple	Y	Unknown
40	*H*. *syriacus* “Minsygrbl1”	Single	Blue	Y	France
41	*H*. *syriacus* “Speciosus”	Double	White	Y	Unknown
42	*H*. *syriacus* “Kakapo”	Double	Pink	Y	Unknown
43	*H*. *syriacus* “Lucy”	Double	Purple	N	Unknown
44	*H*. *syriacus* “Coelestis”	Single	Pink	Y	Unknown
45	*H*. *syriacus* “Minrosa”	Single	Pink	Y	Unknown
46	*H*. *syriacus* f. *elegantissixnus*	Double	Pink	Y	China

**Table 5:** Details of the10SSR primers.

Name	Repeat	Upstream (5′–3′)	Downstream (5′–3′)
HP0001	(TCA)5	TGCCGGAACAAAGGACTCTC	GAATCGCAGGTGGTGGAGAA
HP0002	(GAT)6	CACGCCCTCCAGGAATCTAC	TTCTCAGGTAATGCGGCTGG
HP0003	(CTT)6	ACGGAAGCAAAATCGTTGTCt	TGCTGGAACTTCTGTCGGAC
HP0010	(CAG)5	CAACAGTTGCAGCAGTCACC	GACTGTTGCTGCACCATTGG
HP0011	(CAC)5	CACCACCAATGTCGATGGGA	ACTTGCAGATGGAGGTTGGG
HP0012	(CAG)5	ACCAGAAGAGCTTGGGATGC	AGTGATGCCATTGAGTCTTGGT
HP0015	(AAT)5	GAGGCAGCTTCAAGTTTGGC	CCGGGCCTAAGTTCCCATTT
HP0018	(GAG)5	TCGAGTGGGAGGAAGTGGAT	GAACAAAACCTCCCACCCCA
HP0038	(AG)6	AGAAGAACGCAAGGAGAGGA	TGGAGAACCAGGTCCAGACA
HP0055	(AT)6	CTTCCTTACAGCACGAGCCT	CCCCCACTAGGCCGGATATA

## Data Availability

All the data relevant to this manuscript are available on request from the corresponding author.
